# Supplementary material for: Tropheryma whipplei Colonization in Adults and Children: A Prospective Study
Source: Microorganisms. 2024 Jul 9;12(7):1395. doi: 10.3390/microorganisms12071395 (PMC11279100; doi:10.3390/microorganisms12071395)
Supplement: Supplementary file 1 [file microorganisms-12-01395-s001.zip › microorganisms-3065787-supplementary.pdf]

Table S1. Primer/probe sets of rt-PCRs

Target	Primers/probe	Reference
Cryptosporidium spp.	For 5′-ATGAGCGGGTAACGGGGAAT-3′<br>Rev 5′-CCAATTACAAAACCAAAAAGTCC-3′<br>Probe CY5.5 5′-CGCGCCTGCTGCCTTCTTTAGATG-3′-BHQ	DOI: 10.1099/jmm.0.2008/001461-0
Entamoeba histolytica	For 5′-ATTGTCGTGGCATCCTAACTCA-3′<br>Rev 5′-GCGGACGGCTCATTATAACA-3′<br>Probe 5′-FAM-TCATTGAATGAATTGGCCATTT-3′-MGB	[doi: 10.1046/j.1360-2276.2003.01145.x]
Entamoeba dispar	For 5′-ATTGTCGTGGCATCCTAACTCA-3′<br>Rev 5′-GCGGACGGCTCATTATAACA-3′<br>Probe 5′-HEX-TTACTTACATAAATTGGCCACTTTG-3′-MGB	[doi: 10.1046/j.1360-2276.2003.01145.x]
Giardia intestinalis	For 5′-GACGGCTCAGGACAACGGTT-3′<br>Rev 5′-TTGCCAGCGGTGTCCG-3′<br>Probe 5′-CY55-CCCGCGGCGGTCCCTGCTAG-3′-BHQ	[doi: 10.1016/S0890-8508(03)00057-4]
Dientamoeba fragilis	For 5′-CAACGGATGTCTTGGCTCTTTA-3′<br>Rev 5′-TTGCCAGCGGTGTCCG-3′<br>Probe 5′-HEX-CAATTCTAGCCGCTTAT-3′-MGB	[doi: 10.1016/j.mcp.2007.05.006]
Blastocystis spp.	For 5′-GGTCCGGTGAACACTTTGGATTT-3′<br>Rev 5′-CCTACGGAAACCTTGTTACGACTTCA-3′<br>Probe 5′-FAM-TCGTGTAAATCTTACCATTTAGAGGA-3′-MGB	[doi: 10.1128/JCM.00007-12]
T. whipplei First PCR	For 5′-TGTTTTGTACTGCTTGTAACAGGATCT-3′<br>Rev 5′-TCCTGCTCTATCCCTCCTATCATC-3′<br>Probe 5′-ROX-AGAGATACATTTGTGTTAGTTGTTACA-3′-BHQ	[doi: 10.2217/fmb-2018-0347]

T. whipplei<br>Second PCR	For 5′-TGAGTGATGGTATGTCTGAGAGATATGT-3′<br>Rev 5′-TCCATAACAAAGACAACAACCAATC-3′<br>Probe 5′-FAM-AGAAGAAGATGTTACGGGTTG-3′-TAMRA	[doi: 10.2217/fmb-2018-0347]
PhHV-1	For 5′-GGGCGAATCACAGATTGAATC-3′<br>Rev 5′-GCGGTTCCAAACGTACCAA-3′<br>Probe 5′-Cy5-TTTTTATGTGTCCGCCACCATCTGGATC-3′-BHQ	[doi: 10.1016/S1386-6532(02)00197-X]
